# Mammalian cell-free protein expression promotes the functional characterization of the tripartite non-hemolytic enterotoxin from Bacillus cereus

**DOI:** 10.1038/s41598-020-59634-8

**Published:** 2020-02-19

**Authors:** Franziska Ramm, Srujan Kumar Dondapati, Lena Thoring, Anne Zemella, Doreen Anja Wüstenhagen, Hendrik Frentzel, Marlitt Stech, Stefan Kubick

**Affiliations:** 1 Fraunhofer Institute for Cell Therapy and Immunology (IZI), Branch Bioanalytics and Bioprocesses (IZI-BB), Am Mühlenberg 13, 14476 Potsdam, Germany; 2 Freie Universität Berlin, Institute of Chemistry and Biochemistry – Biochemistry, Takustr. 6, 14195 Berlin, Germany; 3 German Federal Institute for Risk Assessment, Department of Biological Safety, Max-Dohrn-Str. 8-10, 10589 Berlin, Germany; 4 Faculty of Health Sciences, joint Faculty of the Brandenburg University of Technology Cottbus – Senftenberg, the Brandenburg Medical School Theodor Fontane and the University of Potsdam, Potsdam, Germany

**Keywords:** Membrane biophysics, Expression systems, Pathogens

## Abstract

*Bacillus cereus* is increasingly recognized as an opportunistic pathogen causing local and systemic infections. The causative strains typically produce three pore-forming enterotoxins. This study focusses on the tripartite non-hemolytic enterotoxin (Nhe). Until today, studies have tried to elucidate the structure, complex formation and cell binding mechanisms of the tripartite Nhe toxin. Here, we demonstrate the synthesis of the functional tripartite Nhe toxin using eukaryotic cell-free systems. Single subunits, combinations of two Nhe subunits as well as the complete tripartite toxin were tested. Functional activity was determined by hemolytic activity on sheep blood agar plates, planar lipid bilayer measurements as well as cell viability assessment using the MTT assay. Our results demonstrate that cell-free protein synthesis based on translationally active eukaryotic lysates is a platform technology for the fast and efficient synthesis of functionally active, multicomponent toxins.

## Introduction

Foodborne diseases caused by bacterial pathogens play an important role in the worldwide health-care system. *Bacillus cereus (B*. *cereus)* is a ubiquitously distributed bacterial pathogen, which can cause two kinds of foodborne diseases - the diarrhoeal and the emetic^1^. The emetic syndrome is caused by a heat, acid and protease stable peptide toxin called cereulide, which is produced in toxic amounts in food at high bacterial counts. The diarrhoeal symptoms are a consequence of the effect of heat, acid and protease sensitive enterotoxins, which are produced in the human gut after consumption of food contaminated with high numbers of *B*. *cereus* spores (usually ≥10^5^ cfu/g of food)^2^. To date, three major enterotoxins from *B*. *cereus* have been identified which can be classified as pore-forming toxins. Cytotoxin K (CytK) is a single-protein toxin, while the so called non-hemolytic enterotoxin (Nhe) and hemolysin BL (Hbl) are tripartite toxins^1^. *B*. *cereus* strains often produce multiple toxins simultaneously and the genetic prerequisite for producing Nhe, the *nheABC* operon, is most likely present in all *B*. *cereus* strains^3^.

Since the discovery of Nhe in 1996^4^ its structure as well as its functional activity including the pore-formation, have been investigated. Previous studies have demonstrated the importance of the three subunits NheA, NheB and NheC for complex and pore-formation. Initial findings by Lund and Granum (1996)^4^ did not show hemolytic effects of this protein which resulted in naming this protein the non-hemolytic enterotoxin. In contrast to that, later work by Fagerlund *et al*.^5^ demonstrated that hemolytic activity is also detectable for this protein and it is linked to the formation of pores. Whilst initial work discussed the necessity of all three domains for cell binding and cytotoxicity^6^, later work demonstrated an interplay of NheB and NheC prior to the binding of NheA^7^. Cytotoxic activity is not only influenced by a specific membrane-binding order but is also affected by the quantity of the single subunits present. To display maximum functional activity and cytotoxicity, Nhe subunits must interact at a 10:10:1 NheA:NheB:NheC molar ratio^6^. The pore-forming characteristics were first described in 2008, as the combination of all three subunits formed conductance pores measured by planar lipid bilayer recordings^5^. The interaction of NheB with NheC prior to the construction of a full pore including NheA has been subsequently widely discussed^8,9^.

New methods for the characterization of bacterial toxins are required in order to develop diagnostic tools for their detection. In this context, cell-free protein synthesis (CFPS) has emerged as a rapid and efficient method for the synthesis and functional characterization of toxins^10–12^. CFPS is based on translationally active cell lysates, thereby representing an alternative to conventionally used *in vivo* protein production^13^. A major advantage of CFPS is the possibility of synthesizing ‘difficult-to-express’ proteins like toxins or membrane proteins^12,14,15^. By using cell-free systems for protein synthesis, neither cell viability is impaired nor do cellular barriers limit the translation of larger proteins^13,16^. Proteinaceous toxins can be synthesized within a few hours when using cell-free protein synthesis and since an open system is used, the synthesis can be adapted for each individual toxin of interest. In recent years, first studies have shown the efficiency of CFPS for the synthesis of proteinaceous toxins. Apoptosis-inducing toxins such as the pierisin-like protein^10^ as well as hemolysins^11,12,17^ have been successfully synthesized in cell-free systems. Here, we demonstrate the cell-free synthesis of the functional tripartite Nhe toxin. For the first time, we show that eukaryotic cell-free protein synthesis systems offer a way to synthesize functionally active, multicomponent toxins in a fast and efficient manner.

## Material and Methods

### Cell-free protein synthesis

Cell-free synthesis reactions were performed using translationally active lysate derived from Chinese hamster ovary cells (CHO-K1) as previously described^18,19^. Protein synthesis was conducted in coupled transcription/translation reactions in a final volume of 20 to 80 μl. Plasmids, each encoding one of the three subunits of the Nhe-complex, were designed according to Brödel *et al*.^20^. These plasmids were used either separately, in combinations of two plasmids or as a combination of all three plasmids required for forming the tripartite toxin. Additionally, a no template control (NTC) consisting of a translation mixture without the addition of a DNA template was used as a background control. Genes encoding each subunit were obtained by *de novo* gene synthesis (Biocat GmbH). Gene sequences derived from *Bacillus cereus* strain ATCC 14579 were used: AE016877.1:1765248–1766408 for NheA, AE016877.1:1766446–1767654 for NheB and AE016877.1:1767762–1768841 for NheC. Sequences were cloned into the pUC57–1.8 K vector backbone and directly used as templates.

Two different reaction formats were used in this study. First, batch-based reactions were incubated in a thermomixer (Eppendorf) for 3 h at 30 °C and 500 RPM if not stated otherwise. Cell-free synthesis reactions were composed of 40% (v/v) translationally active lysate supplemented with HEPES-KOH (pH 7.6, f.c. 30 mM, Carl Roth GmbH), sodium acetate (f.c. 100 mM, Merck), Mg(OAc)_2_ (f.c. 3.9 mM, Merck), KOAc (f.c. 150 mM, Merck), amino acids (complete 100 μM, Merck), spermidin (f.c. 0.25 mM; Roche), Dithiothreitol (DTT, 2.5 mM, Life technologies GmbH) and energy regenerating components including creatine phosphokinase (f.c. 0.1 mg/ml, Roche), creatine phosphate (20 mM, Roche), ATP (1.75 mM, Roche) and GTP (0.3 mM, Roche). To enable DNA transcription during cell-free protein synthesis, 1 U/μl T7 RNA polymerase, 0.3 mM of UTP (Roche) and CTP (Roche) and 0.1 mM of the cap analogue m7G(ppp)G (Prof. Edward Darzynkiewicz, Warsaw University, Poland) were added to the reaction. Additionally, PolyG primer (IBA) was supplemented at a final concentration of 12 µM. As needed, cell-free protein synthesis reactions were supplemented with radioactive ^14^C-leucine (f.c. 50 μM, specific radioactivity 66.67 dpm/pmol, Perkin Elmer) for radio-labeling of produced proteins, in order to allow for further analysis by autoradiography and liquid scintillation counting.

Secondly, cell-free reactions were conducted using continuous-exchange cell free (CECF) reaction formats as described previously^15^. CECF-reactions were performed in commercially available dialysis devices (SCIENOVA) and incubated in a thermomixer (Eppendorf) for 24 h, at 27 °C and 600 RPM if not stated otherwise. Two mixtures, namely the reaction mixture and the feeding mixture, were individually prepared. Reaction mixtures were composed similar to batch-based reactions (see above). Additionally, PolyG was added at a final concentration of 4.5 µM and Mg(OAc)_2_ was added at a final concentration of 18.5 mM. The feeding mixture was composed of HEPES-KOH (f.c. 30 mM, pH 7.6), Mg(OAc)_2_ (f.c. 3.9 mM), KOAc (f.c. 150 mM), amino acids (complete 100 µM f.c.), spermidine (f.c. 0.25 mM), energy regenerating components (f.c. 1.75 mM ATP, 0.3 mM GTP), CTP (f.c. 0.3 mM), UTP (f.c. 0.3 mM) and the cap analogue G(ppp)G (f.c. 0.33 mM). Further, the caspase inhibitor AC-DEVD-CMK (30 µM; Promega) and sodium azide (f.c. 0.02%, Merck) were added to both reaction and feeding mixture. Radioactive ^14^C-leucine (f.c. 50 μM, specific radioactivity 10 dpm/pmol) for radio-labeling of *de novo* produced proteins was added to the reaction to allow for subsequent quantitative and qualitative analyses of synthesized proteins by liquid scintillation counting and autoradiography.

After incubation, the translation mixture (TM) was centrifuged (16,000 × g, 10 min, 4 °C) resulting in the supernatant (SN) containing the soluble subunits.

### Analysis of radio-labeled proteins

Total protein yields of cell-free synthesized proteins were determined by incorporation of ^14^C-leucine and subsequent precipitation by hot trichloro acetic acid (TCA, Carl Roth GmbH). Briefly, 3 µl aliquots of TM and SN were mixed with 3 ml of 10% TCA/2% casein (Carl Roth GmbH) hydrolysate solution and incubated at 80 °C for 15 min. Following a 30 min incubation on ice, ^14^C-labeled proteins were transferred to membrane filters (VWR) using a vacuum filtration system (Hoefer). Filters were washed with 5% TCA and dried with acetone twice to remove non-incorporated ^14^C-leucine. Finally, dried filters were placed in 3 ml scintillation cocktail, incubated for at least 1 h and measured by liquid scintillation counting using the LS6500 Multi-Purpose scintillation counter (Beckman Counter). The total protein yield for coexpressed subunits was estimated using the sum of the molecular weight and the sum of the number of leucines of all expressed subunits according to the following equations:

$${Protein}\,{yield}\,[\frac{\mu {g}}{{mL}}]=\frac{{scintillation}\,{counts}[\frac{{dpm}}{{mL}}]\ast {molecular}\,{weight}[\frac{\mu {g}}{{pmol}}]}{{specific}\,\text{radioactivity}\,[\frac{{dpm}}{{pmol}}]\ast {number}\,{of}\,{leucines}}$$

$$Specific\,radioactivity\,[\frac{dpm}{pmol}]=\frac{stock\,concentration\,of\,14C\,leucine\ast specific\,radioactivity\,of\,14C\,leucine\,stock}{Total\,concentration\,of\,leucine}$$

Sodium dodecyl sulfate polyacrylamide gel electrophoresis (SDS-PAGE) using precast gels (NuPAGE, 10%Bis-Tris, Life technologies) was performed to confirm the molecular mass of radio-labeled, cell-free synthesized proteins. 3 µl aliquots of TM and SN were precipitated in cold acetone (Carl Roth GmbH), incubated on ice for at least 15 min and the mixture was centrifuged at 4 °C and 16,000 × g for 10 min. The supernatant was discarded, and the protein pellet was dried at 45 °C for a minimum of 40 min in a thermomixer. Dried protein pellets were resuspended in 20 µl LDS sample buffer (NUPAGE LDS sample buffer supplemented with 50 mM DTT (Applichem GmbH)). As a final preparation step, synthesized protein samples were heated to 70 °C for 10 min. Proteins were separated on precast NUPAGE SDS-PAGE gels for 35 min at 185 V and were stained with Coomassie blue solution (SimplyBlue SafeStain, Life technologies). Subsequently, gels were dried on Whatman paper for 70 min at 70 °C (Unigeldryer 3545D, Uniequip). Dried gels, harboring radio-labeled proteins were exposed to phosphor screens for a minimum of three days. Finally, radio-labeled proteins were visualized using a phosphor imager system (Typhoon TRIO+ Imager, GE Healthcare). Autoradiographs were processed using the ImageQuant TL Software.

### Analysis of fluorescently-labeled proteins

As an alternative labeling technique, pre-charged tRNAs were utilized. Commercially available pre-charged tRNA recognizing the phenylalanine codon was chemically aminoacylated to lysine harbouring the fluorescent dye Bodipy-TMR. A batch-based cell-free synthesis reaction as described above was performed in the presence of Bodipy-TMR-GAA (f.c. 2 µM, Biotech rabbit). Following the reaction, 5 µl of the SN fraction were precipitated with ice cold acetone and separated on NUPAGE SDS-PAGE gels as described above. Fluorescently labeled proteins were visualized using the Typhoon TRIO+ Imager (GE Healthcare) using an excitation of 544 nm and an emission of 570 nm.

### Duopath cereus enterotoxins (Merck)

To demonstrate that cell-free expressed Nhe toxins are comparable to the native bacterial enterotoxins, a commercially available test for the detection of the Nhe subunit NheB, the Duopath Cereus Enterotoxins (Merck), was performed. This test is commonly applied to bacterial culture supernatants to confirm the production of Nhe (and also Hbl) by *B*. *cereus* isolates. For the testing of cell-free expressed tripartite Nhe, 20 µl of the supernatant fraction were diluted in 130 µl PBS. The solution was applied to the lateral flow assay and incubated for 30 min. PBS and an NTC were used as controls. Subsequently, an image was taken for verification.

### Cell-based expression of Nhe tripartite toxin

To produce Nhe containing culture supernatant, *Bacillus cereus* (s.l.)-strain BfR-BA-00963 was grown for 6 h in BHI at 30 °C and 150 rpm using a shaking incubator. After incubation, the culture was centrifuged for 20 min at 4,000 g and 4 °C. The supernatant was filter sterilized (pore size 0.2 µm) and kept frozen until further use. To confirm the production of active Nhe, 150 µl of the culture supernatant was applied to the Duopath assay.

The presence of the genes *nheA*, *nheB* and *nheC* and the absence of the genes *hblC*, *hblD*, *hblA* and *ces* as well as *cytK-1* and *cytK-2* in strain BfR-BA-00963 was proven by multiplex PCR according to Guinebretiere *et al*.^21^ and Wehrle *et al*.^22^.

### Planar lipid bilayer electrophysiology for measuring the Nhe activity

Lipid bilayers were formed from 1,2‐diphytanoyl‐sn‐glycero‐3‐phosphocholine (DPhPC) and cholesterol (CHO) (Avanti Polar Lipids, Albaster, AL, USA). Lipids were dissolved in octane (Sigma Aldrich) in a ratio of 90:10 (DPhPC:CHO) at a concentration of 10 mg/mL. Concentrations of 1 M KCl (Sigma Aldrich (Fluka)), 10 mM HEPES (Sigma Aldrich), buffered at pH 7.45 were used as an electrolyte. Polydispersed poly ethylene glycol (polyPEG‐1500, Mw = 1400–1600 g/mol) (FLUKA, Sigma Aldrich) was dissolved in the electrolyte solution to a concentration of 10 mg/mL and 2 μL of this solution was added to the 180 μL electrolyte in the measurement chamber for recordings. The experimental setup was described previously^12,23^. A single channel amplifier (EPC‐10, HEKA Electronic Dr. Schulze GmbH, Lambrecht, Germany) was connected to the multiplexer electronics port of the Orbit16 system. Recordings were done at a sampling rate of 50 kHz with a 10 kHz Bessel filter. Data were analyzed with Clampfit (Molecular Devices, Sunnyvale, CA, USA).

### Hemolytic activity assessment

The hemolytic activity of cell-free produced Nhe toxin was assessed using precast blood agar plates containing 5% sheep blood (VWR). A total volume of 10 µl of *de novo* synthesized toxin (TM or SN) was directly spotted onto the blood agar plate. To suppress microbial growth, erythromycin and gentamycin (f.c. 50 µg/ml) were added to each sample. After 24 h of incubation at 37 °C, hemolytic zones were visualized and documented. 0.25% Triton-X 100 detergent solution served as a positive control. To probe defined toxin concentrations, protein yields were determined by TCA-precipitation as described under “Analysis of radio-labeled proteins” and defined concentrations were calculated.

### Cell-based toxicity studies

For toxicity studies, the MTT cytotoxicity assay (Boster’s MTT Cell Proliferation Assay Kit, Boster Biological Technology Co., Ltd.) was used. CaCo_2_ cells (ATCC) were cultured in Minimum Essential Medium (MEM) supplemented with 20% fetal bovine serum (Merck, Biochrom GmbH), 1% non-essential amino acids (Merck, Biochrom GmbH) and 1% Penicillin/Streptomycin (Merck, Biochrom GmbH). Cells were split every 5–7 days according to standard protocol using 0.25% trypsin/0.02% EDTA (Merck, Biochrom GmbH). The cell cultures were maintained in a cell culture incubator at 37 °C and 5% carbon dioxide (CO_2_) atmosphere. In this study, 100 μl of a 100,000 cells/ml CaCo_2_ cell suspension were added to each well of a 96 well microplate (f.c. 10,000 cells/well). Subsequently, 10 μl SN fraction harbouring the cell-free synthesized tripartite toxin or negative controls was added to the wells. Cells were incubated for 24 h in a cell incubator at 37 °C with 5% CO_2_. Cell viability was determined by the addition of 3-(4,5-dimethylthiazol-2-yl)-2,5-diphenyltetrazolium bromide (MTT). The absorbance was measured at 570 nm and 630 nm using the Mithras LB 943 (Berthold). The cell viability was calculated accordingly:

$$Cell\,viability\,[ \% ]=\frac{OD\,570-OD\,630\,of\,test\,substance}{OD\,570-OD\,630\,of\,0\,nM\,test\,substance}\ast 100$$

Each sample was analyzed in triplicates. As negative controls untreated cells, where only cell culture medium was added, and an NTC synthesized in a batch reaction were used. The latter was diluted and treated in the same manner as the proteinaceous toxin used in the assay and applied in a volume equivalent to the toxin.

Toxin concentrations of the radio-labeled SN fraction were measured by TCA-precipitation (see above) and, consequently, assumed for non-labeled SN fractions as both reactions were prepared simultaneously. Serial dilutions of cell-free synthesized Nhe toxin ranging from 0.1 nM to 1 nM were tested.

## Results

### Synthesizing functionally active Nhe using cell-free protein synthesis

Cell-free protein synthesis offers a rapid and efficient way to synthesize ‘difficult-to-express’ proteins such as membrane proteins^12,14,15^ but also opens new possibilities for studying multicomponent proteins. Up to now, there mainly have been studies of multicomponent proteins in prokaryotic cell-free systems^24–26^ and lysates derived from human cell-lines^27,28^. Here, we demonstrate the cell-free synthesis and functional characterization of the Nhe tripartite toxin and its subunits using lysate derived from cultured CHO cells and show the self-assembly process of a bacterial toxin in a CHO-based cell-free system.

**Figure 1:**
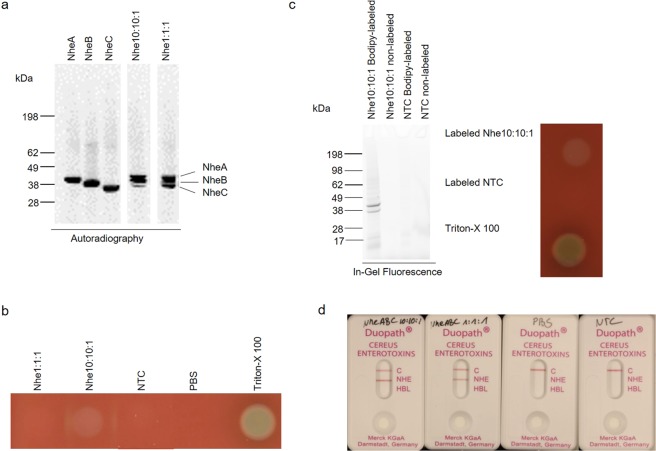
Cell-free synthesis of functional Nhe. Nhe subunits A, B and C were synthesized in CHO lysates either separately or coexpressed to form the tripartite toxin. Autoradiograph showing ^14^C-leucine labeled Nhe subunits and Nhe tripartite toxin when synthesized using molar plasmid concentrations of 10:10:1 and 1:1:1 ratios (**a**). Hemolytic activity of the Nhe tripartite toxins was assessed on 5% sheep blood agar plates (**b**). Bodipy-labeled Nhe toxin was assessed by in-gel fluorescence and functional activity was assessed by 5% sheep blood agar plates (**c**). *De novo* synthesized Nhe tripartite toxins were tested in the Duopath lateral flow assay (Merck). NTC reactions and PBS were used as controls. A line at C shows a control line indicating a valid test result (**d**).

Initial experiments were conducted to identify the capacity of CFPS to synthesize each protein subunit individually and to produce a tripartite toxin. Previous studies have shown that the three individual subunits of the tripartite toxin have to be present in a defined molar ratio of 10:10:1 [A:B:C] to provide maximum functional activity^6^. In our study, we added the plasmids encoding the Nhe subunits A, B and C individually and in different molar plasmid ratios to cell-free reactions. Single subunits as well as their combination were successfully synthesized as indicated by autoradiography (Fig. 1a) and liquid scintillation measurement (Supplementary Fig. 1a). The combination of the plasmids in a 10:10:1 molar ratio led to hemolytic activity while a 1:1:1 molar ratio showed no activity (Fig. 1b). Neither the expression of each single subunit individually nor expression of combinations of two subunits was sufficient to induce hemolysis (Supplementary Fig. 1b,c, respectively). Further combinations of plasmid concentrations were probed in the cell-free system and the products were analyzed for hemolytic activity. The addition of twice the molar plasmid concentration of a single subunit or the addition of twice the molar plasmid ratio of NheA and B as compared to NheC was tested. While all combinations showed defined protein bands by autoradiography (Supplementary Fig. 2a), none of the additionally tested concentrations showed hemolytic activity (Supplementary Fig. 2b). As the coexpression of all three subunits simultaneously in a 10:10:1 molar plasmid ratio led to the active tripartite toxin, we wanted to determine whether the addition of NheA and NheB plasmid at higher ratios than 2:2:1 led to hemolytic activity. Hence, molar plasmid ratios of 7:7:1, 6:6:1 and 5:5:1 were compared to 10:10:1 and 1:1:1 [A:B:C]. Again, the autoradiograph clearly showed defined protein bands (Supplementary Fig. 2c) and all reactions showed hemolytic activity (Supplementary Fig. 2d), which suggests that NheA and NheB generally have to be present in excess over NheC.

#### NheC’s role in complex formation

To further investigate the role of NheC in complex formation, the hemolytic activity of the Nhe tripartite toxin was studied when adding varying molar plasmid ratios of NheC to a 10:10 molar plasmid ratio of NheA and NheB (Supplementary Fig. 3). In a first experiment, NheC was supplemented at a molar plasmid ratio ranging from 1 to 10 and showed that only a combination of all three plasmids in a 10:10:1 or 10:10:2 molar plasmid ratio resulted in hemolytically active protein (Supplementary Fig. 3b). Next, a combination of NheABC in a 10:10:2.1 molar plasmid ratio was investigated showing no hemolytic activity (Supplementary Fig. 4a). Nhe tripartite toxin expressed in 10:10:1, 10:10:1.5, 10:10:1.7, 10:10:1.9, 10:10:2.1 and 10:10:2.3 molar plasmid ratio was studied for hemolytic activity. As shown in Supplementary Fig. 4b, Nhe toxin synthesized in a 10:10:1.5, 10:10:1.7 and 10:10:1.9 molar plasmid ratio was hemolytically active but 10:10:2.1 and 10:10:2.3 were not. The hemolytic activity of Nhe expressed in 10:10:1, 10:10:1.9, 10:10:2.0 and 10:10:2.1 molar plasmid ratios was investigated two additional times in the same lysate and in a different CHO lysate batch. In accordance with previous experiments, Nhe expressed in a molar plasmid ratio of 10:10:2.1 was not active (Supplementary Fig. 4c,d). These data show that NheC can be supplemented at a maximum molar plasmid ratio of 2 in a coupled cell-free synthesis reaction. Additionally, the autoradiograph from Supplementary Fig. 3a was semi-quantitatively analyzed, as explained in the Supplementary Methods, in order to assess the protein expression of each individual subunit. This analysis showed that when synthesizing Nhe in a 10:10:1 molar plasmid ratio manner about 55% of the total synthesized protein was NheA, about 34% was NheB and about 10% was NheC resulting in a protein ratio of 11.6:7.2:2.2 (Supplementary Table 1). With increasing supplemented NheC plasmid, as shown in Supplementary Fig. 3a, the protein ratio shifts to a higher protein yield of synthesized NheC. To further study the protein yield of NheC in the final complex and describe the threshold when a complex was not formed any more, autoradiographs of Supplementary Fig. 4a,b were semi-quantitatively analyzed. These analyses showed that when NheC reached a total protein level of four, the coexpressed subunits were not hemolytically active anymore (Supplementary Table 3). The data shown here, demonstrate that the synthesized protein ratio does not completely correspond to the supplemented molar plasmid ratio. Nonetheless, it could be shown that when synthesizing the Nhe tripartite toxin in a 10:10:1 manner, NheA and NheB were synthesized in excess over NheC. Further experiments used a molar plasmid ratio of 10:10:1 if not stated otherwise.

#### Fluorescent labeling of Nhe

As an open system, cell-free protein synthesis allows for the addition of diverse supplements to the reaction mixture thereby offering versatile possibilities for protein modification. One alternative to radio-labeling is the use of fluorescently labeled dyes. In this study, Bodipy-TMR was used to label the Nhe toxin at phenylalanine positions. As shown in Fig. 1c, the labeling reaction showed intensely labeled protein bands by in-gel fluorescence (Fig. 1c). Two distinct labeled protein bands were detected by in-gel fluorescence corresponding to NheA and NheB. These reactions also showed hemolytic activity on sheep blood agar plates (Fig. 1c).

#### Duopath Cereus Enterotoxin detection

Next, cell-free synthesized toxins were analyzed by the commercially available Duopath Cereus Enterotoxins (Merck) detection method. *De novo* synthesized Nhe tripartite toxin produced in molar plasmid ratios of 10:10:1 and 1:1:1 were tested. As expected, both Nhe tripartite toxin reactions tested positive while PBS and the NTC showed no detectable NheB (Fig. 1d). As a result, we could show that cell-free synthesized Nhe tripartite toxin is functionally active as demonstrated by hemolytic activity on sheep blood agar plates and detectable by the commercially available Duopath. Single subunits as well as the coexpression of two or three subunits can be analyzed using cell-free protein synthesis. Nonetheless, as we used molar plasmid ratios for cell-free synthesis reactions, the precise ratio of the synthesized protein subunits in the assembled tripartite toxin is still elusive.

### Optimizing batch-based cell-free protein synthesis reactions

**Figure 2:**
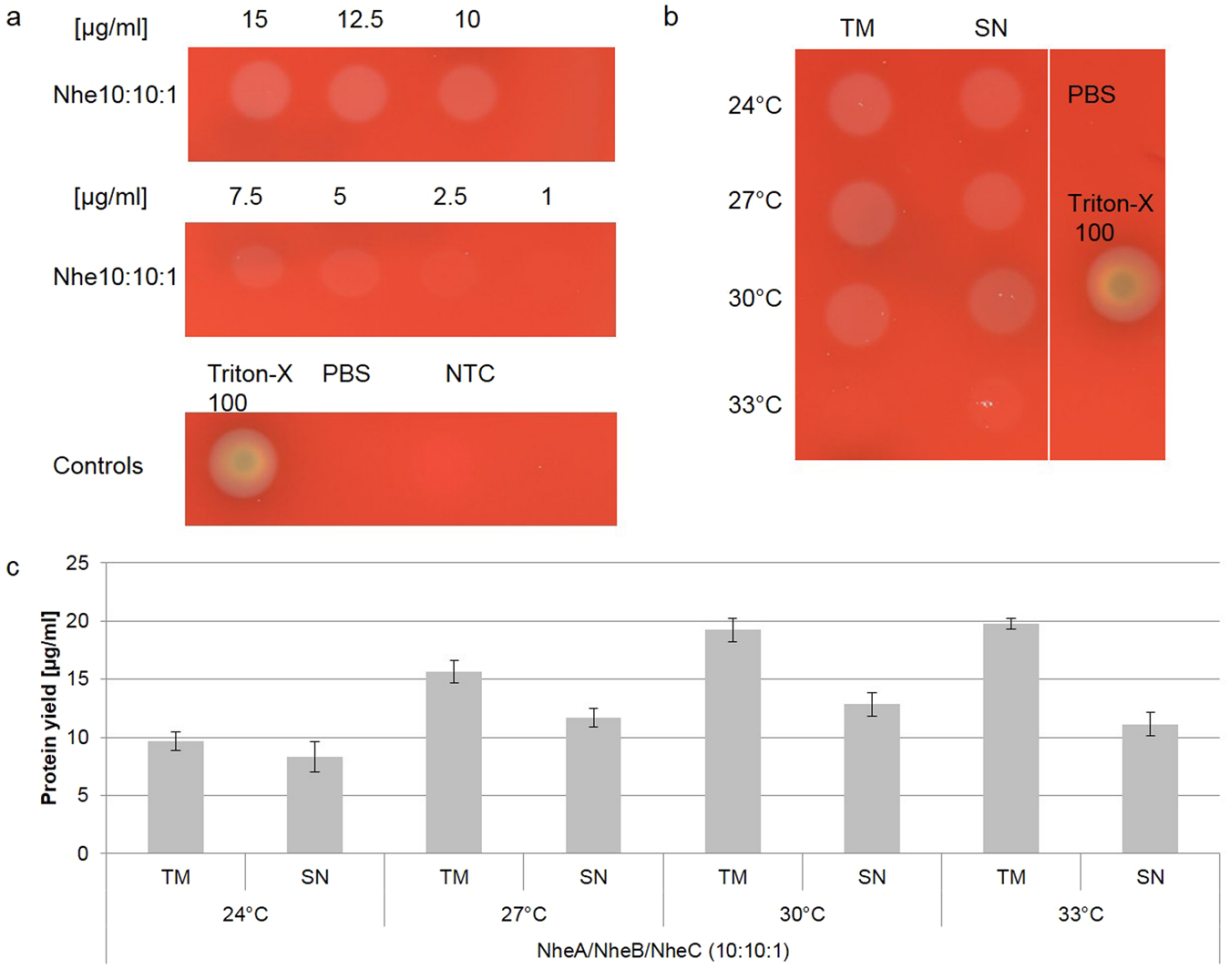
Optimization of batch-based cell-free reactions. Coexpressed Nhe subunits A, B and C were synthesized in a CHO lysate at a molar plasmid ratio of 10:10:1. The hemolytic activity was assessed on 5% sheep blood agar plates. In order to identify initial hemolytic activity a serial dilution of the total reaction mixture containing *de novo* synthesized Nhe tripartite toxin was spotted on the blood agar plate. Concentrations are shown in µg/ml (**a**). Different temperatures were assessed for cell-free toxin synthesis. Again, hemolytic activity analysis (**b**) and liquid scintillation counting (**c**) were performed for both the reaction mixture (TM) and the supernatant (SN). Total protein yields of *de novo* synthesized Nhe toxin were analyzed by liquid scintillation. The total protein yield for coexpressed subunits was estimated using the sum of the molecular weight and the sum of the number of leucines of all expressed subunits. Standard deviations were calculated from triplicate analysis.

As the usage of a 10:10:1 [A:B:C] molar plasmid ratio resulted in the production of functional toxin, a serial dilution of *de novo* synthesized Nhe tripartite toxin was spotted onto blood agar plates to analyze the amount of toxin required to induce hemolysis. Initial hemolytic activity was detected at a concentration of 5 µg/ml of Nhe tripartite toxin (Fig. 2a). Increasing toxin concentrations resulted in more intense hemolytic areas (Fig. 2a).

Further experiments aimed to optimize batch-based reactions to result in the highest possible protein yields while maintaining the toxic character of the protein. As each toxin might need individual synthesis conditions, we aimed to identify the optimal conditions for the tripartite Nhe toxin complex. To identify the optimal temperature for batch-based cell-free reactions, the reaction mixture was incubated at 24 °C, 27 °C, 30 °C and 33 °C for 3 h. At 33 °C no hemolysis was detected on the blood agar plates while incubation temperatures of 24 °C, 27 °C and 30 °C showed identical patterns of hemolysis (Fig. 2b). Total protein yields increased with rising temperatures up to 30 °C (Fig. 2c). Consequently, further batch-based experiments were conducted at 30 °C. We aimed to identify the optimal duration of the cell-free reaction using incubation times of 1.5 h, 2 h, 2.5 h, 3 h, 3.5 h and 4 h (Supplementary Fig. 5). With increasing time surpassing 3 h, the total protein yield increased (Supplementary Fig. 5a) but the hemolytic activity decreased (Supplementary Fig. 5b). For further experiments, a 3 h incubation time was chosen as the yield was acceptable for further functional activity assessment and the hemolysis on the blood agar plates indicated clear activity.

**Figure 3:**
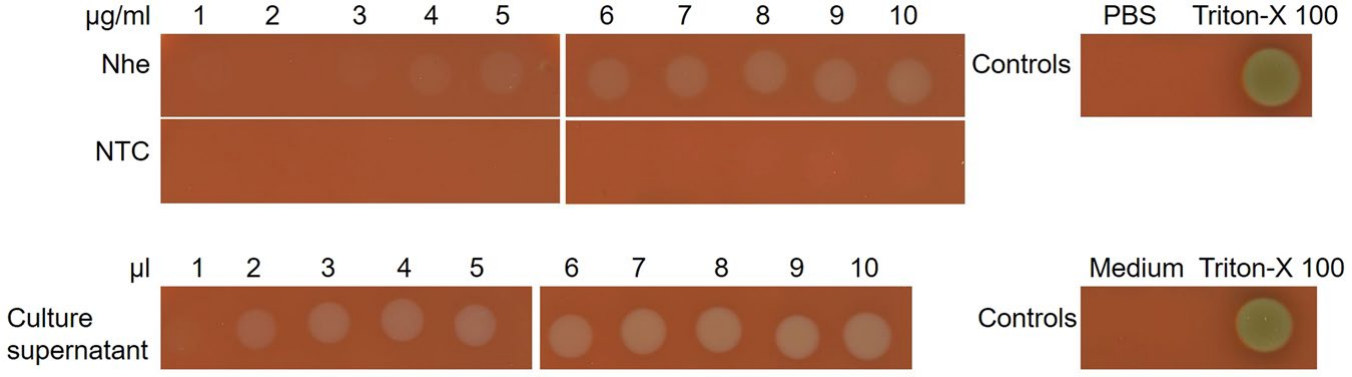
Hemolytic activity of cell-free synthesized Nhe tripartite toxin compared to bacterial Nhe toxin. Coexpressed Nhe subunits A, B and C were synthesized in a CHO lysate at a molar plasmid ratio of 10:10:1. The hemolytic activity was assessed on 5% sheep blood agar plates. In order to identify initial hemolytic activity, a serial dilution in PBS of the total reaction mixture containing *de novo* synthesized Nhe tripartite toxin was spotted on the blood agar plate. Concentrations are shown in µg/ml. Different dilutions of Nhe containing culture supernatant from *Bacillus cereus* (s.l.)-strain BfR-BA-00963 in culture medium were spotted onto 5% sheep blood agar plates to compare the functional activity of cell-free expressed to cell-based expressed Nhe toxin. PBS and Triton-X 100 served as controls for cell-free expression and culture medium and Triton-X 100 were controls for cell-based expression.

Using these optimized synthesis conditions, the hemolytic activity of cell-free synthesized Nhe tripartite toxin was compared to Nhe toxin expressed in the *Bacillus cereus* (s.l.)-strain BfR-BA-00963. Therefore, a serial dilution ranging from 1 to 10 µg/ml of cell-free synthesized Nhe protein was spotted onto 5% sheep blood agar plates. 10 µl of each individual mixture harboring 1 to 10 µl of Nhe containing culture supernatant was spotted onto 5% sheep blood agar plates (Fig. 3). Culture supernatant was diluted in culture medium BHI. The size of the hemolytic ring on the blood agar plates induced by the cell-free synthesized toxin was compared to the lytic activity of the culture supernatant as described in the Supplementary Methods. This semi-quantitatively comparison showed that cell-free synthesized Nhe at a concentration of 10 µg/ml results in a similar hemolytic area as 7–8 µl of culture supernatant containing Nhe (Supplementary Table 2).

### Optimizing CECF-based cell-free protein synthesis reactions

**Figure 4:**
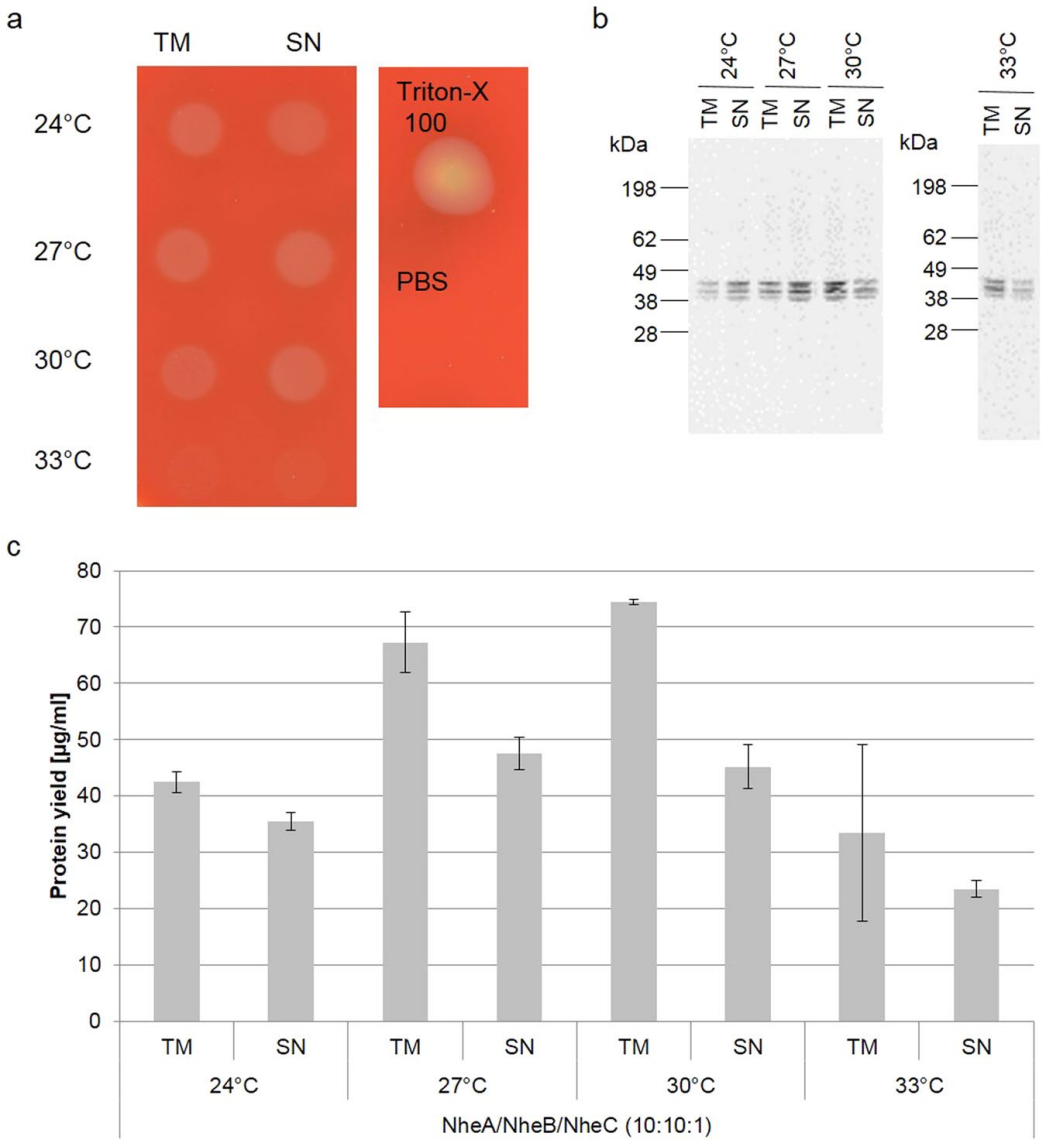
Synthesis optimization in CECF reactions. CECF-synthesis of Nhe toxin under different conditions was optimized with respect to optimal hemolytic activity on sheep blood agar plates. Coexpressed Nhe subunits A, B and C were synthesized in a CHO lysate at different temperatures (24 °C, 27 °C, 30 °C, 33 °C) using a 10:10:1 molar plasmid ratio. Hemolytic activity was assessed for the tripartite toxin (**a**). Autoradiograph showing ^14^C-leucine labeled, Nhe subunits synthesized at the indicated temperature (**b**). Total protein yield was analyzed by liquid scintillation measurement for TM and SN fractions. The total protein yield for coexpressed subunits was estimated using the sum of the molecular weight and the sum of the number of leucines of all expressed subunits. Standard deviations were calculated from triplicate analysis (**c**).

Cell-free reactions can be conducted in a dialysis-based reaction mode, also termed continuous-exchange cell-free (CECF) mode, to increase total protein yields^15,29,30^. CECF-based reactions were performed to synthesize proteinaceous toxins over a time period of 24 h and the optimal temperature was determined as for the batch-based reactions. Incubation temperatures of 24 °C, 27 °C and 30 °C resulted in cell-free synthesized tripartite toxin displaying hemolytic activity (Fig. 4a). As also observed in the batch-based experiments, no hemolysis was observed at a temperature of 33 °C (Fig. 4a). Autoradiography confirmed protein bands for the three different Nhe subunits at all four temperatures (Fig. 4b). In the CECF-mode maximum protein yields were obtained for 27 °C and 30 °C (~67 µg/ml and ~74 µg/ml, respectively). Total protein yields were lower at 24 °C and 33 °C (~42 µg/ml and ~33 µg/ml, respectively, Fig. 4c). The temperature gradient ranging from 24 to 33 °C identified 27 °C as an optimum (Fig. 4a–c). While reactions incubated at 27 °C and 30 °C showed hemolytic activity in TM and SN fractions (Fig. 4a) and both temperatures resulted in maximum total protein yields (Fig. 4c), more soluble protein was found at 27 °C: 70% of the protein was soluble at 27 °C while 60% was soluble at 30 °C.

### Pore-formation assessed by planar lipid bilayer recordings

**Figure 5:**
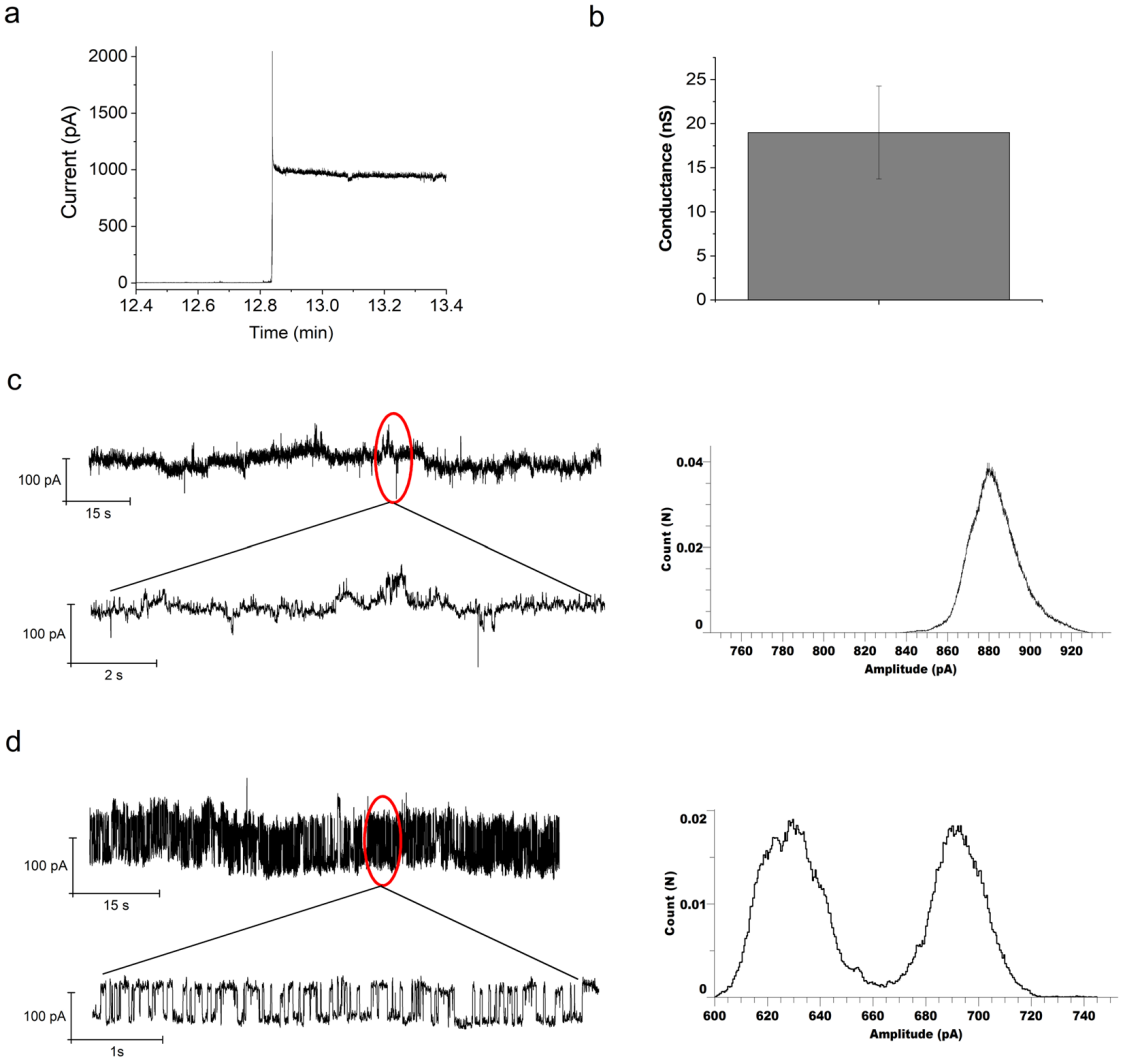
Current response from the planar bilayer in the presence of Nhe10:10:1 tripartite toxin. Conductance jump due to insertion of the toxin (**a**). Average conductance jump at +60 mV from multiple measurements (n = 6) (**b**). Continuous recordings of current response at +60 mV in the presence of Nhe10:10:1 toxin. Magnifying a small portion of the pore current shows a stable response. Histogram of the current response measured at +60 mV (**c**). Continuous recordings of Nhe10:10:1 current response in the presence of PEG 1500 at +60 mV. Magnifying a small portion of the response shows a stable single channel activity with transitions between open and closed states. Histogram of the current response measured at +60 mV (**d**). Buffer: 10 mM HEPES, 1 M KCl pH 7.45. Planar lipid bilayer formed from 10 mg/ml DPhPC: cholesterol (90:10) in octane.

Planar lipid bilayer electrophysiology was used to study the pore forming properties of Nhe 10:10:1 toxin using membranes formed from DPhPC:Chol (90:10). Figure 5 shows the pore currents electrically recorded after the insertion of the Nhe10:10:1 tripartite toxin at +60 mV. Control experiments were performed with the Nhe1:1:1 reaction mixture to check the characteristic currents. We observed that there is a conductance jump upon pore insertion. In the case of Nhe10:10:1, we observed multiple pores with different conductances. Large pores with an average conductance jump of 18 nS +/− 6 nS at 1 M KCl conditions were only observed in the case of Nhe10:10:1 (Fig. 5a). Additionally, small conductances in the range of 5 nS +/− 1.5 nS as well as single channel activity of 0.5 nS were detected. The average conductance in the case of Nhe1:1:1 was small (3.3 nS +/− 1 nS) and unstable. When a small part of the Nhe10:10:1 recorded trace is magnified as shown in Fig. 5c, a stable current without any transition is observed, which resembles an open pore. The all-time histogram of the whole current trace from Fig. 5c showed a broader single peak corresponding to the single current level without any fluctuation. After recording the currents over time to ensure the stability of the pore, PEG1500 was added to the bilayer to check the single channel activity via polymer blocking. In the presence of PEG molecules, the current baseline level decreased from 880 pA to 690 pA with time and showed single channel activity with transitions between two current levels (630 and 690 pA). An all point histogram of the whole current trace from Fig. 5c showed two peaks of similar intensity corresponding to the equal distribution of current levels as observed in the continuous trace.

### Cell-based cytotoxicity of the cell-free synthesized Nhe toxin on CaCo_2_ cells

**Figure 6:**
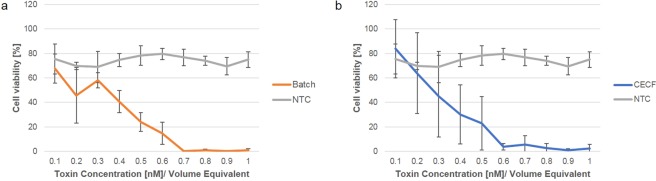
MTT cytotoxicity assay. Cell-free synthesized Nhe was assessed for its cytotoxic effect on CaCo_2_ cells using the MTT cytotoxicity assay. The Nhe tripartite toxin was synthesized in batch (**a**) and CECF mode. (**b**) An NTC was used as a negative control. A serial dilution of Nhe ranging from 0.1 nM to 1 nM was tested for both synthesis modes. Standard deviations were calculated from triplicate samples of three independent experiments.

Enterotoxins from *B*. *cereus* are formed in the small intestine subsequent to germination of spores and cell outgrowth in close proximity to the epithelial cells where the enterotoxins act^2^. Previous studies have tested the cytotoxic effects of *B*. *cereus* enterotoxins on a variety of cell lines including intestinal tissue derived cells^31^. Here, we investigated the cytotoxic effects of the Nhe tripartite toxin on the human colorectal cancer cell line CaCo_2_. The viability of CaCo_2_ cells was assessed 24 h after the addition of the Nhe tripartite toxin. A serial dilution of batch and CECF synthesized toxin showed increasing cytotoxic effect with increasing toxin concentration (Fig. 6a,b). Nhe toxin synthesized in the batch-based mode reduced cell viability at approximately the same concentrations as CECF synthesized toxin. Batch-based Nhe resulted in complete cell death at 0.7 nM whereas 0.6 nM of CECF synthesized Nhe induced complete cell death (Fig. 6a,b, respectively).

## Discussion

Bacterial toxins spark the interest of health care officials as well as scientists. *Bacillus cereus* bacterial strains produce three medically and scientifically relevant pore-forming enterotoxins. This study focusses on the non-hemolytic enterotoxin (Nhe). As a toxin consisting of three different subunits, Nhe offers a versatile set of possibilities for future applications as the individual subunits can each be mutated and analyzed. In this context, Nhe might be an interesting model protein for subunit interaction studies. Prior studies have focused on the cell binding effects of Nhe, its *in vivo* production and cytotoxicity^6,7,31,32^. This is the first study to show the cell-free protein synthesis of Nhe. In our study, the tripartite Nhe toxin demonstrated hemolytic as well as cytotoxic activity (Figs. 1 and 6, respectively) and its pore-formation was confirmed by planar lipid bilayer recordings (Fig. 5).

Pore-forming toxins, such as the enterotoxins from *B*. *cereus*, cause virulence as they perforate membrane structures. Hence, the detailed characterization of rarely described pore-forming toxins is essential in terms of diagnostic and medical interest as well as for single molecule analyses. The mechanism of action whereby Nhe acts on the human gastrointestinal tract remains to be elucidated. Previous *in vivo* studies showed that a pre-assembly of NheC and NheB takes place during initial pore formation while NheA is added later for the completion of the full pore^7–9^. Here we show planar lipid bilayer current responses for the Nhe tripartite toxin (Fig. 5a–d). In the presence of PEG molecules, the current baseline level reduced from 880 pA to 690 pA with time and showed a single channel activity with transitions between two current levels (Fig. 5c). This typical behavior is observed in the cytolysin like pores^5,33^. The large and stable conductances, which were regularly observed only in the case of Nhe10:10:1, are likely due to the insertion of a complete toxin complex which upon fusion to the planar lipid bilayer causes the current jump. The smaller conductance values might be due to insertion of partially formed pores or activity caused by individual complexes between two subunits^33,34^. These data add to previous data suggesting a self-assembly process of the pore.

In contrast to classical cell-based protein production, cell-free systems offer a high flexibility regarding the synthesis of proteins. Cell-free protein synthesis is a time-saving method that can easily be adapted for each individual toxin and for each individual toxin subunit. In this way, toxin syntheses can be efficiently applied to a wide range of downstream applications. As shown here, individual toxin subunits and combinations of several domains at defined molar plasmid ratios can be analyzed (Supplementary Figs. 1 and 2) and incubation temperature (Figs. 2 and 4) as well as incubation time (Supplementary Fig. 5) can be adjusted according to the protein’s needs. This work showed that an incubation temperature of 33 °C did not result in a hemolytically active toxin complex (Figs. 2 and 4). As a matter of fact, this may indicate a temperature sensitive protein being part of the complex. Previous data showed decreased growth rates and enterotoxin production at temperatures exceeding 30 °C^35^. This work indicates that no hemolytically active enterotoxin complex can be formed at temperatures exceeding 30 °C.

Due to the open character of cell-free systems, exogenous components can easily be applied. This feature enables the incorporation of non-canonical amino acids that can be fluorescently, or otherwise, labeled^36,37^ which is an alternative to radio-labeling. Our study shows for the first time that labeling of the tripartite Nhe toxin was possible (Fig. 1c). Fluorescently labeled proteins can be used for a variety of applications as each subunit can be labeled individually. With the introduction of a defined fluorescent label in each subunit of a tripartite toxin, interaction studies for the characterization of the assembly complex might be possible. The labeled toxin can be supplemented to a cell line, such as the CaCo_2_ cell line in case of the Nhe toxin. Subsequently, the labeled toxin could be monitored as it enters the cell and as it is processed. This can be used to identify the toxin’s target within the cell as well as its degradation process. In case of multi-component pore-forming toxins, when the different subunits of the toxin are labeled individually, the process of membrane insertion as well as the assembly of the complete pore could be monitored. The toxin can be synthesized in a cell-free manner and easily provided for the development of such assays. Further, labeling techniques can be used in cell-free systems enabling a variety of toxin detection applications. Apart from that, labeling techniques can be used for the variation of the diameter of the pore. Insertion of a fluorescent label might widen the diameter of the pore, which would lead to different current responses when characterizing the pore-forming toxin using electrophysiological measurements. Thinking of nanopores and their diverse applications such as sequencing of DNA and RNA as well as characterizing polymers and proteins^38,39^, fluorescently labeled pores offer the possibility to develop versatile types of nanopores with different diameters in order to sequence and characterize diverse molecules.

Cell-free protein synthesis might also be used as a platform technology to identify critical toxic concentrations of proteinaceous toxins. Previous work has shown that cell-free protein synthesis can be performed based on PCR-products^10,40^. This technique offers the possibility to produce toxic proteins and truncated toxins in a cloning-free manner, thereby avoiding the generation of genetically modified organisms. Reagents inhibiting toxin effects can easily be analyzed and hence, a variety of antibodies including commercially available as well as newly developed antibodies and potential treatments can be screened. Typically, bacterial strains differ tremendously as they harbor resistances and mutations. Sequence variances of enterotoxins produced from different bacterial strains can be difficult to detect and a further functional characterization is rather challenging. As a high number of different mutants can be analyzed in parallel in cell-free systems, health relevant toxins could be identified easily. Such mutation analyses are of diagnostic relevance as resistant bacterial strains and more potent toxins evolve. Expression PCR offers a fast and versatile method to insert point mutations and deletions of known active centers. Future experiments should include a screening of different mutants in each subunit of the Nhe tripartite toxin to analyze their effects on the hemolytic activity as well as complex formation. This shows that cell-free protein synthesis enables high throughput screening of potential treatments as well as high throughput toxicity screening of a variety of proteinaceous toxin mutants. Using an eukaryotic cell lysate facilitates functional testing as no purification of the synthesized protein is necessary in contrast to prokaryotic lysates. Eukaryotic lysates do not contain endotoxins, which is a prerequisite for functional testing in cell-based assays. In previous studies it was shown that certain eukaryotic lysates, in particular the CHO-based cell-free system as well the insect based *Spodoptera frugiperda* cell-free system *Sf*21, contain microsomal vesicles^18,41^. These vesicles mimic membrane like structures, which are necessary for the production of membrane proteins. Pore-forming toxins that do not assemble themselves as the Nhe, might need a membranous structure for the correct folding of the protein or, when multiple subunits are present, for the assembly process. In contrast to standard prokaryotic cell-free systems no additional supplementation of membranous structures such as nanodiscs^42^ is needed as these endogenous structures are already present in this particular eukaryotic cell-free system.

Taken together, our study demonstrates that cell-free protein synthesis presents an advanced technology for the fast production and functional characterization of unknown toxins. Single structures, protein combinations as well as complex protein mixtures can be screened for their toxicity. In the case of Nhe, similar characteristics to *in vivo* derived data concerning functionality and complex-formation were shown.

## Supplementary information

Supplementary Information.

## Data Availability

All relevant data are within the paper and its Supporting Information files.
