# Supplementary material for: Mammalian cell-free protein expression promotes the functional characterization of the tripartite non-hemolytic enterotoxin from Bacillus cereus
Source: Sci Rep. 2020 Feb 19;10:2887. doi: 10.1038/s41598-020-59634-8 (PMC7031377; doi:10.1038/s41598-020-59634-8)
Supplement: Supplementary file 1 — Supplementary Information. [file 41598_2020_59634_MOESM1_ESM.pdf]

Supplementary Information

Mammalian cell-free protein expression promotes the functional characterization of the tripartite non-hemolytic enterotoxin from Bacillus cereus

Franziska Ramm[1,2], Srujan Kumar Dondapati[1], Lena Thoring[1], Anne Zemella[1], Doreen Anja Wüstenhagen[1], Hendrik Frentzel[3], Marlitt Stech[1] and Stefan Kubick[1, 4]

Affiliations

[1]Fraunhofer Institute for Cell Therapy and Immunology (IZI), Branch Bioanalytics and Bioprocesses (IZI-BB), Am Mühlenberg 13, 14476 Potsdam, Germany

[2] Freie Universität Berlin, Institute of Chemistry and Biochemistry – Biochemistry, Takustr. 6, 14195 Berlin, Germany

[3] German Federal Institute for Risk Assessment, Department of Biological Safety, Max-Dohrn-Str. 8-10, 10589 Berlin, Germany

[4] Faculty of Health Sciences, joint Faculty of the Brandenburg University of Technology Cottbus – Senftenberg, the Brandenburg Medical School Theodor Fontane and the University of Potsdam

Supplementary Information

Supplementary Material

Semi-quantitative analysis of autoradiographs

Autoradiograph was analyzed using ImageQuantTL software (GE Healthcare Life Sciences). Each lane shows three distinct bands representing NheA (top), NheB (middle) and NheC (bottom). The intensity of each individual band in each lane was measured resulting in a band volume. For each band the volume was normalized to the number of leucines present in the single protein subunit according to the following calculation:

$$Normalized\ band\ volume = \frac{Band\ volume}{Total\ number\ of\ leucines\ in\ all\ subunits} * Number\ of\ leucines\ in\ single\ subunit$$

Total number of leucines in all subunits: 91
Number of leucines in NheA: 34
Number of leucines in NheB: 31
Number of leucines in NheC: 26

Further, the percentage of each synthesized subunit was calculated according to:

$$Percentage\ of\ expressed\ protein[\%] = \frac{Normalized\ Band\ volume\ for\ subunit}{Total\ Band\ Volume\ for\ all\ three\ subunits} * 100$$

To compare the supplemented molar plasmid ratio with the synthesized protein ratio for the three subunits, the following calculation was applied:

$$Protein\ ratio\ for\ each\ subunit = \frac{Normalized\ Band\ volume\ for\ subunit}{Total\ Band\ Volume\ for\ all\ three\ subunits} * Number\ of\ plasmid\ units$$

The number of plasmid units was calculated for each synthesis. A synthesis in a 10:10:1 manner has 21 (10+10+1) plasmid units.

Semi-quantitative analysis of hemolytic area

To determine the diameter of the hemolytic area on blood agar plates, a defined length of 1 cm was photographed. This defined length as well as the lytic areas on the blood agar plates were measured using ImageJ (National Institutes of Health). The measured pixels were translated to a defined length in cm according to the following calculation:

$$Length\ (cm) = \frac{Measured\ pixels}{Measured\ pixels\ of\ 1\ cm\ defined\ length}$$

Supplementary Results

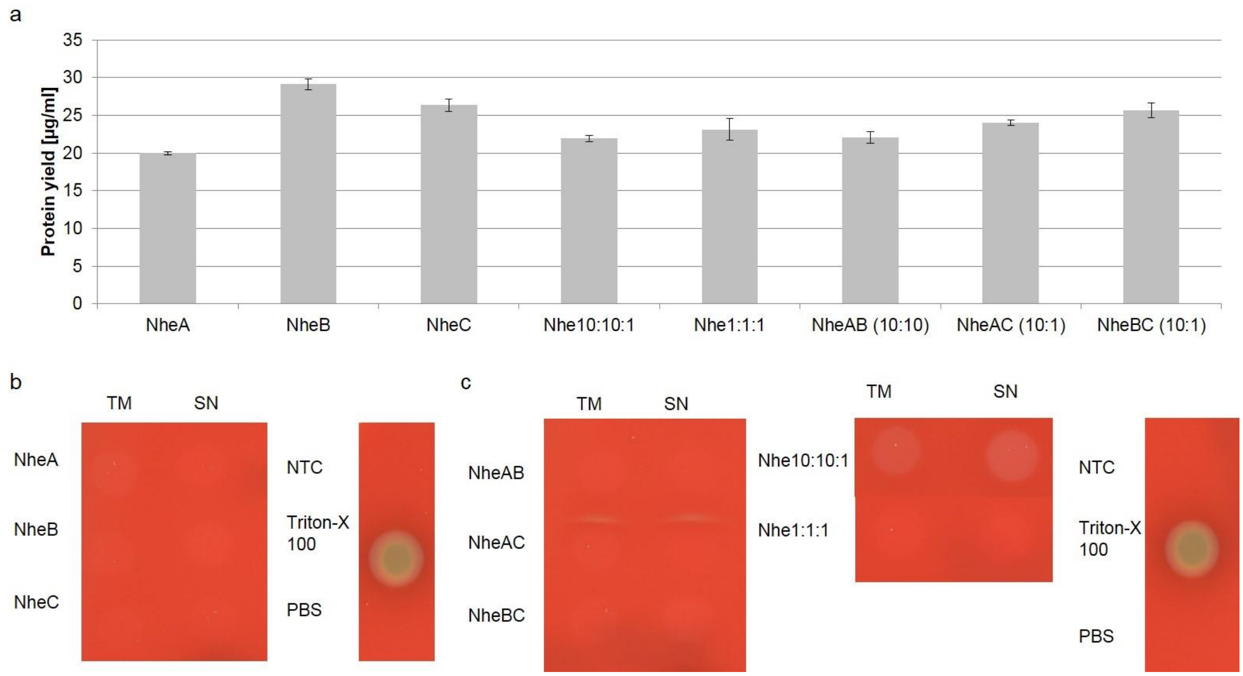

Supplementary Figure 1: Synthesis of the Nhe subunits and the tripartite toxin. Nhe single subunits (f.c. 60 µg/ml plasmid concentration), Nhe A, B and C coexpressed in either 10:10:1 or 1:1:1 molar plasmid ratios for tripartite toxins and combining two subunits (NheAB 10:10, NheAC 10:1 and NheBC 10:1 molar plasmid ratio) were synthesized in a CHO lysate. Total yields of *de novo* synthesized Nhe subunits were analyzed by liquid scintillation counting. The total protein yield for coexpressed subunits was estimated using the sum of the molecular weight and the sum of the number of leucines of all expressed subunits. Standard deviations were calculated from triplicate analysis (a). Hemolysis was assessed on 5% sheep blood agar plates to test functional activity for single subunits (b) and their combination (c). Hemolytic activity was assessed for the translation mixture (TM) and the supernatant (SN).

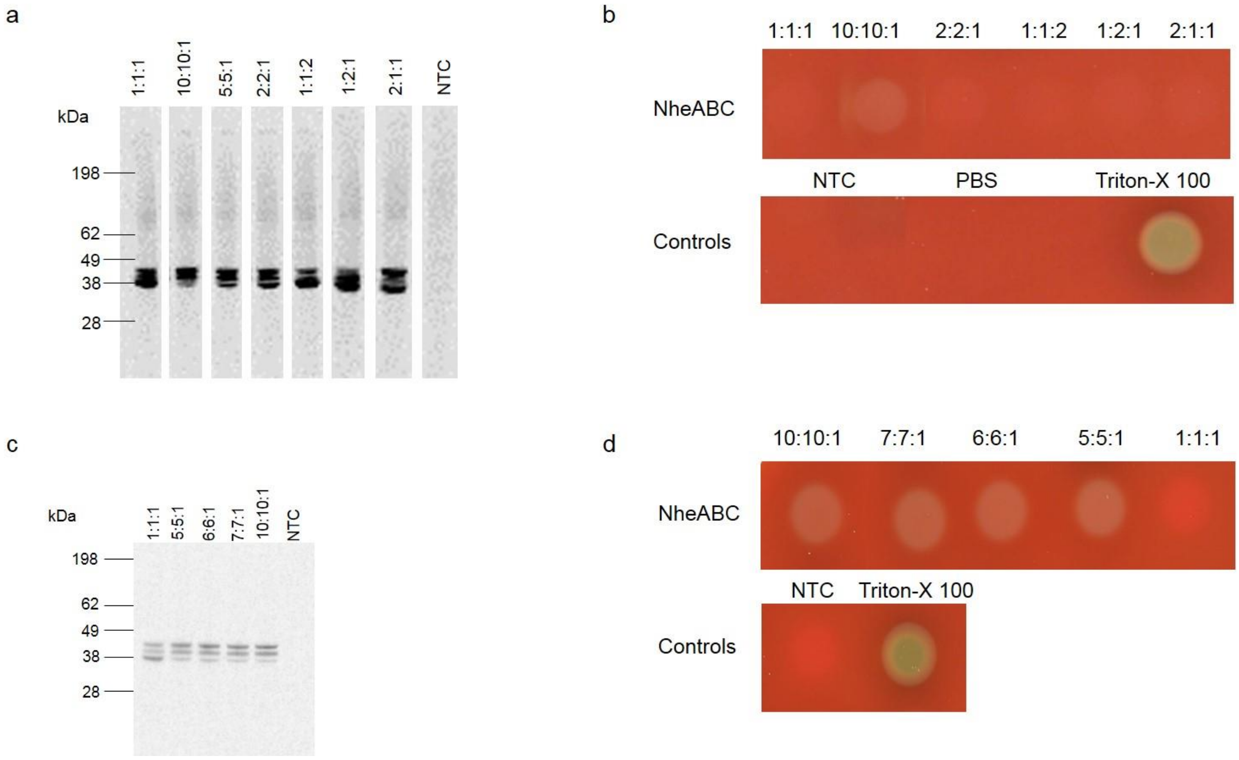

Supplementary Figure 2: Synthesis and hemolytic activity of different plasmid molar ratios for Nhe tripartite toxin. Coexpressed Nhe subunits A, B and C were synthesized in CHO lysate and their hemolytic activity was assessed on 5% sheep blood agar plates. PBS and a non-template control (NTC) were used as negative controls and Triton-X 100 as a positive control. Autoradiograph showing ^{14}C-leucine labeled Nhe tripartite toxin when synthesized in 1:1:1, 10:10:1, 2:2:1, 1:1:2, 1:2:1 and 2:1:1 [A:B:C] molar plasmid ratios (a). Hemolytic activity of the reaction mixtures in A was assessed (b). Autoradiograph showing ^{14}C-leucine labeled Nhe subunits when synthesized in 10:10:1, 7:7:1, 6:6:1, 5:5:1 and 1:1:1 [A:B:C] molar plasmid ratios from a further experiment (c). Hemolytic activity of the reaction mixtures in c was assessed (d).

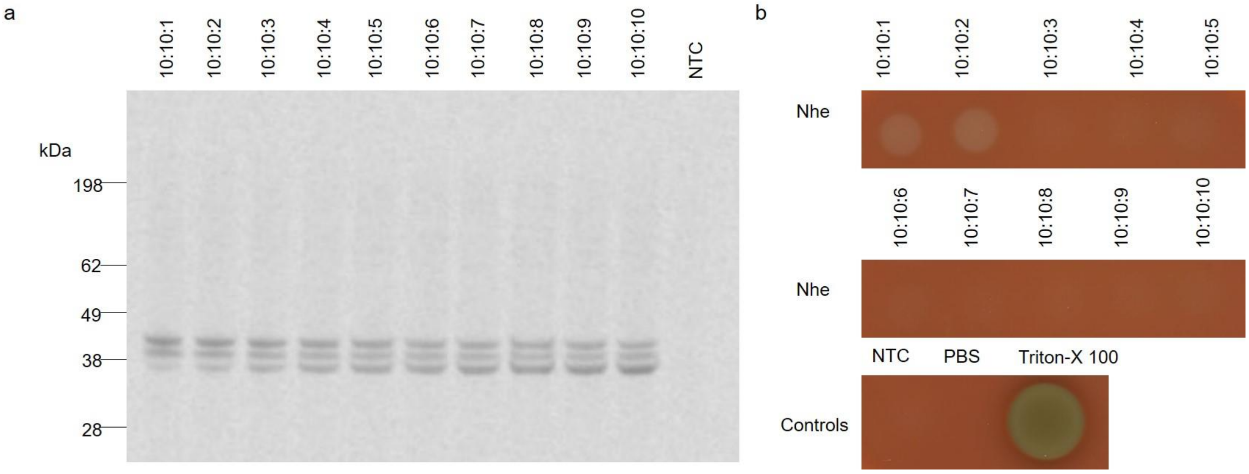

Supplementary Figure 3: Synthesis and hemolytic activity based on different plasmid molar ratios. Coexpressed Nhe subunits A, B and C were synthesized in CHO lysate and their hemolytic activity was assessed on 5% sheep blood agar plates. PBS and a non-template control (NTC) were used as negative controls and Triton-X 100 was used as a positive control. Nhe A and B were synthesized in a 10:10 molar plasmid ratio at all settings while NheC was varied from a 1 to 10 molar plasmid ratio. Autoradiograph showing ^{14}C-leucine labeled Nhe tripartite toxins (a). Hemolytic activity of 8 µg/ml of the reaction mixtures in A was assessed (b).

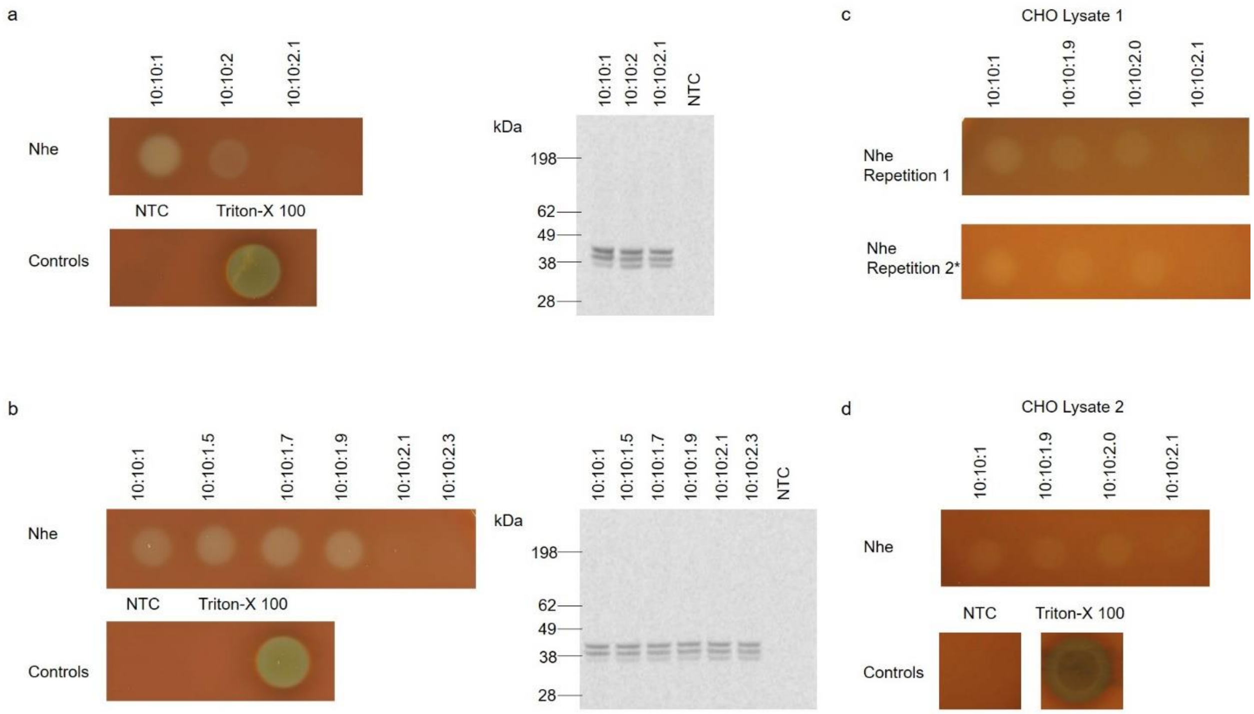

Supplementary Figure 4: NheC's role for complex formation. Coexpressed Nhe subunits A, B and C were synthesized in CHO lysate and their hemolytic activity was assessed on 5% sheep blood agar plates. A non-template control (NTC) was used as negative control and Triton-X 100 was used as a positive control. Nhe A and B were synthesized in a 10:10 molar plasmid ratio at all settings while NheC was varied from a 1 to 2.3 molar plasmid ratio. Hemolytic activity of three reaction mixtures, Nhe expressed in [10:10:1], [10:10:2] and [10:10:2.1] was assessed on sheep blood agar plates and via autoradiography (a). Nhe expressed in [10:10:1], [10:10:1.5], [10:10:1.7], [10:10:1.9], [10:10:2.1] and [10:10:2.3] molar plasmid ratios was assessed on sheep blood agar plates and via autoradiography (b). Nhe expression in [10:10:1], [10:10:1.9], [10:10:2.0] and [10:10:2.1] molar plasmid ratios was repeated two additional times and analyzed for hemolytic activity (c). *Contrast of blood agar plate for Repetition 2 was increased. Nhe expression in [10:10:1], [10:10:1.9], [10:10:2.0] and [10:10:2.1] molar plasmid ratios was repeated in a further CHO lysate (d).

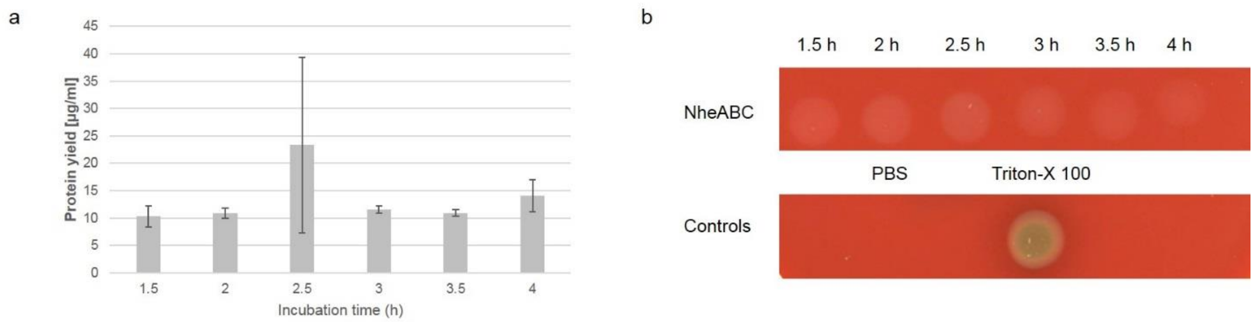

Supplementary Figure 5: Analysis of cell-free incubation time for Nhe synthesis. Coexpressed Nhe subunits A, B and C were synthesized in CHO lysate at different incubation times and the hemolytic activity of the supernatant (SN) was assessed on sheep blood agar plates. Total protein yields of *de novo* synthesized Nhe subunits were analyzed by liquid scintillation counting. The total protein yield for coexpressed subunits was estimated using the sum of the molecular weight and the sum of the number of leucines of all expressed subunits. Standard deviations were calculated from triplicate analysis (a). Hemolytic activity of the Nhe toxin produced at different incubation times was assessed (b). PBS and a non-template control (NTC) were used as negative controls and Triton X 100 as a positive control.

Supplementary Table 1: Semi-quantitative analysis of autoradiograph from Supplementary Figure 3a.

Lane	Band No	Protein	Band Volume	Normalized	%	Protein Ratio
Lane 1 (10:10:1)	1	NheA	1200847.60	448668.33	55.30	11.61
	2	NheB	816693.38	278214.23	34.29	7.20
	3	NheC	295607.21	84459.20	10.41	2.19
Lane 2 (10:10:2)	1	NheA	1110788.63	415019.93	54.16	11.92
	2	NheB	675573.34	230140.37	30.03	6.61
	3	NheC	423967.45	121133.56	15.81	3.48
Lane 3 (10:10:3)	1	NheA	958753.36	358215.54	50.60	11.64
	2	NheB	587061.81	199988.09	28.25	6.50
	3	NheC	524233.44	149780.98	21.16	4.87
Lane 4 (10:10:4)	1	NheA	859536.32	321145.44	45.27	10.87
	2	NheB	587286.48	200064.63	28.20	6.77
	3	NheC	658609.15	188174.04	26.53	6.37
Lane 5 (10:10:5)	1	NheA	895649.27	334638.19	45.11	11.28
	2	NheB	530700.48	180788.08	24.37	6.09
	3	NheC	792169.72	226334.21	30.51	7.63
Lane 6 (10:10:6)	1	NheA	763868.79	285401.53	40.26	10.47
	2	NheB	576818.40	196498.58	27.72	7.21
	3	NheC	794362.44	226960.70	32.02	8.32
Lane 7 (10:10:7)	1	NheA	821884.54	307077.74	39.91	10.78
	2	NheB	526828.26	179468.97	23.33	6.30
	3	NheC	990010.07	282860.02	36.76	9.93
Lane 8 (10:10:8)	1	NheA	841561.20	314429.46	37.71	10.56
	2	NheB	548179.62	186742.51	22.40	6.27
	3	NheC	1164312.21	332660.63	39.90	11.17
Lane 9 (10:10:9)	1	NheA	771917.88	288408.88	35.29	10.24
	2	NheB	519100.81	176836.54	21.64	6.28
	3	NheC	1231652.57	351900.73	43.06	12.49
Lane 10 (10:10:10)	1	NheA	554359.23	207123.23	24.24	7.27
	2	NheB	645583.89	219924.18	25.73	7.72
	3	NheC	1496443.17	427555.19	50.03	15.01

Supplementary Table 2: Semi-quantitative analysis of hemolytic area.

Protein	µg/ml	Measured Pixels	Calculated length [cm]
Cell-free Nhe	10	120.04	0.69
	9	120.00	0.69
	8	105.17	0.60
	7	102.00	0.59
	6	108.00	0.62
	5	102.00	0.59
	4	108.04	0.62
	3	87.05	0.50
Protein	µl	Measured Pixels	Calculated length [cm]
Culture Supernatant	10	126.04	0.72
	9	114.00	0.66
	8	123.00	0.71
	7	117.00	0.67
	6	105.04	0.60
	5	96.05	0.55
	4	96.00	0.55
	3	93.05	0.54
	2	108.00	0.62
Triton-X 100	10	150.03	0.86
1 cm		174.00	1

Supplementary Table 3: Semi-quantitative analysis of autoradiographs from Supplementary Figure 4.

Lane	Band No	Protein	Band Volume	Normalized	%	Protein Ratio
Lane 1 (10:10:1)	1	NheA	327721.86	122445.53	43.52	9.14
	2	NheB	379371.63	129236.49	45.93	9.65
	3	NheC	103940.94	29697.41	10.55	2.22
Lane 2 (10:10:2)	1	NheA	343134.09	128203.95	45.56	10.02
	2	NheB	315239.5	107389.28	38.17	8.40
	3	NheC	160258.3	45788.09	16.27	3.58
Lane 3 (10:10:2.1)	1	NheA	313117.28	116988.87	48.98	10.82
	2	NheB	225417.44	76790.56	32.15	7.10
	3	NheC	157819.96	45091.42	18.88	4.17
Lane 1 (10:10:1)	1	NheA	227884.74	85143.75	47.61	10.00
	2	NheB	220387.25	75076.98	41.98	8.82
	3	NheC	65201.98	18629.14	10.42	2.19
Lane 2 (10:10:1.5)	1	NheA	228334.79	85311.90	47.34	10.18
	2	NheB	203714.04	69397.09	38.51	8.28
	3	NheC	89282.44	25509.27	14.16	3.04
Lane 3 (10:10:1.7)	1	NheA	195180.5	72924.58	44.93	9.75
	2	NheB	187687.82	63937.61	39.39	8.55
	3	NheC	89096.22	25456.06	15.68	3.40
Lane 4 (10:10:1.9)	1	NheA	195045.9	72874.29	43.57	9.54
	2	NheB	186830.14	69804.67	41.73	9.14
	3	NheC	86064.52	24589.86	14.70	3.22
Lane 5 (10:10:2.1)	1	NheA	343134.09	128203.95	46.12	10.19
	2	NheB	293519.13	99990.03	35.97	7.95
	3	NheC	174265.31	49790.09	17.91	3.96
Lane 6 (10:10:2.3)	1	NheA	187546.65	70072.38	46.24	10.31
	2	NheB	143057.83	48733.99	32.16	7.17
	3	NheC	114564.53	32732.72	21.60	4.82
